# The Role of Mast Cells in the Remodeling Effects of Molecular Hydrogen on the Lung Local Tissue Microenvironment under Simulated Pulmonary Hypertension

**DOI:** 10.3390/ijms252011010

**Published:** 2024-10-13

**Authors:** Dmitrii Atiakshin, Andrey Kostin, Alexander Alekhnovich, Artem Volodkin, Michael Ignatyuk, Ilya Klabukov, Denis Baranovskii, Igor Buchwalow, Markus Tiemann, Marina Artemieva, Nataliya Medvedeva, Tyler W. LeBaron, Mami Noda, Oleg Medvedev

**Affiliations:** 1 RUDN University, 6 Miklukho-Maklaya St, 117198 Moscow, Russia; andocrey@mail.ru (A.K.); alekhnovich_av@pfur.ru (A.A.); volodkin-av@rudn.ru (A.V.); ignatyuk-ma@rudn.ru (M.I.); buchwalow@pathologie-hh.de (I.B.); maminoda39@gmail.com (M.N.); oleg.omedvedev@gmail.com (O.M.); 2 Research Institute of Experimental Biology and Medicine, Burdenko Voronezh State Medical University, 394036 Voronezh, Russia; 3 National Medical Research Radiological Centre of the Ministry of Health of the Russian Federation, Koroleva st. 4, 249036 Obninsk, Russiadoc.baranovsky@gmail.com (D.B.); 4 Institute for Hematopathology, Fangdieckstr. 75a, 22547 Hamburg, Germany; mtiemann@hp-hamburg.de; 5 Faculty of Biology, Lomonosov Moscow State University, Leninskie Gory 1-12, 119234 Moscow, Russia; marinka.artemieva@gmail.com (M.A.); namedved@gmail.com (N.M.); 6 Department of Kinesiology and Outdoor Recreation, Southern Utah University, Cedar City, UT 84720, USA; tylerlebaron@suu.edu; 7 Molecular Hydrogen Institute, Cedar City, UT 84720, USA; 8 Center for Mitochondrial Biology and Medicine, The Key Laboratory of Biomedical Information Engineering of Ministry of Education, School of Life Science, Xi’an Jiaotong University, Xi’an 710049, China; 9 Faculty of Medicine, Lomonosov Moscow State University, Lomonosovsky Prospect 27-1, 119991 Moscow, Russia

**Keywords:** molecular hydrogen, mast cells, pulmonary hypertension, hydrogen inhalations, collagen fibrillogenesis, collagen and elastic fibers, fibrous niches of the local tissue microenvironment, secretory pathways, tryptase, heparin

## Abstract

Molecular hydrogen (H_2_) has antioxidant, anti-inflammatory, and anti-fibrotic effects. In a rat model simulating pulmonary fibrotic changes induced by monocrotaline-induced pulmonary hypertension (MPH), we had previously explored the impact of inhaled H_2_ on lung inflammation and blood pressure. In this study, we further focused the biological effects of H_2_ on mast cells (MCs) and the parameters of the fibrotic phenotype of the local tissue microenvironment. MPH resulted in a significantly increased number of MCs in both the pneumatic and respiratory parts of the lungs, an increased number of tryptase-positive MCs with increased expression of TGF-β, activated interaction with immunocompetent cells (macrophages and plasma cells) and fibroblasts, and increased MC colocalization with a fibrous component of the extracellular matrix of connective tissue. The alteration in the properties of the MC population occurred together with intensified collagen fibrillogenesis and an increase in the integral volume of collagen and elastic fibers of the extracellular matrix of the pulmonary connective tissue. The exposure of H_2_ together with monocrotaline (MCT), despite individual differences between animals, tended to decrease the intrapulmonary MC population and the severity of the fibrotic phenotype of the local tissue microenvironment compared to changes in animals exposed to the MCT effect alone. In addition, the activity of collagen fibrillogenesis associated with MCs and the expression of TGF-β and tryptase in MCs decreased, accompanied by a reduction in the absolute and relative content of reticular and elastic fibers in the lung stroma. Thus, with MCT exposure, inhaled H_2_ has antifibrotic effects involving MCs in the lungs of rats. This reveals the unknown development mechanisms of the biological effects of H_2_ on the remodeling features of the extracellular matrix under inflammatory background conditions of the tissue microenvironment.

## 1. Introduction

The central role of reactive oxygen species (ROS) in triggering inflammation during the onset and progression of multiple diseases is well established [1,2,3]. The pathological overproduction of ROS provokes a persistent state of oxidative stress and, subsequently, fibrosis [4,5,6]. In the local tissue microenvironment, including the lungs, mast cells (MCs) play a key role in regulating the local homeostasis and integrative buffer metabolic environment. Indeed, we previously reported that a reduction in tryptase-containing MCs in the experimental model of pulmonary hypertension in rats [7], which had a single subcutaneous injection of MCT (60 mg/kg in 60% ethyl alcohol) for 21 days, with one group *(n* = 8) inhaling room air while the other group (*n* = 8) inhaled room air with 4% hydrogen. MCs have a wide range of receptors, giving them high sensitivity to various stimuli of a specific tissue microenvironment [8,9,10,11]. These receptors include protein tyrosine phosphatase non-receptor type 3, the expression of which can change significantly in different types of cancer [12]. During activation, MCs can selectively secrete various classes of mediators, cytokines, and chemokines, thereby providing the structure of the immune and stromal landscape of a specific tissue microenvironment, including the one during inflammation [13]. MCs influence tissue microenvironment targets using three classes of mediators: pre-formed; lipid-derived; and a diverse array of cytokines, chemokines, and growth factors synthesized in response to MC stimulation [14,15,16]. MCs are crucial in the development of inflammation, affecting the state of numerous immune and connective tissue cell subpopulations, as well as the extracellular matrix of connective tissue [17,18,19,20,21,22]. From the pathogenetic point of view of the development of fibrotic niches, the interaction between MCs and alveolar type II cells is interesting because it is capable of contributing to the fibroproliferative reaction by secreting growth factors and proinflammatory molecules after damage [23], as well as monocytes and macrophages, which play a key role in the development of fibrosis, including systemic sclerosis-related interstitial lung disease [24].

The small size of molecular hydrogen (H_2_), its low mass, neutral charge, and non-polar nature, combined with a high diffusion rate, allow it to easily penetrate cell biomembranes and diffuse into mitochondria and the nucleus, where it exerts many biological effects [25,26,27]. As demonstrated, H_2_ has therapeutic and preventive effects on various organs due to its antioxidant properties, neutralizing hydroxyl radicals and reducing peroxynitrite levels [28]. Yet, the mechanisms of how H_2_ initiates these biological effects, including anti-inflammatory, anti-apoptotic, neuroprotective, radioprotective, adaptive, and homeostatic effects, have not been specified [26,27,28,29,30,31,32,33]. H_2_ can reduce inflammation, regulate immunocompetent cells, modulate cell death pathways (e.g., apoptosis, autophagy, and pyroptosis) and other signaling pathways, and exert mitochondrial effects [28,33,34,35]. Thus, H_2_ can be considered a promising agent for regulating the integrative buffer metabolic environment of the local tissue microenvironment, primarily the lungs and liver, due to its distribution properties and biological effects. 

Implementing the biological effects of H_2_ on the structures of a specific tissue microenvironment through its impact on MCs, which are likely the target of H_2_, seems pathogenetically validated [36]. To date, several studies related to the effect of H_2_ on the state of MCs have been conducted. In particular, H_2_ leads to decreased migration and secretory activity of MCs and a decreased intensity of inflammation in some organs [34,37,38]. An experiment involving rats demonstrated the positive effects of H_2_ on the regeneration of the skin dermis after burn injury, which was associated with the physiological activity of MCs in the remodeling of the extracellular matrix of connective tissue [39]. However, this is evidently insufficient to reveal the real potential of MCs in mediating the biological effects of H_2_. This study focused on investigating the precise role of MCs in developing biological effects of H_2_ inhalation in the lungs of rats under simulated pulmonary hypertension; in particular, the reduction in the fibrotic phenotype of the local tissue microenvironment though the relationship between the development of fibrosis and the number of MCs is well known [7,40,41,42,43]. At the same time, the precise molecular mechanisms of how MCs are involved in forming the profibrogenic background require further clarification and research, which may shed light on new possibilities of MCs in remodeling the extracellular matrix.

## 2. Results

**Figure 1:**
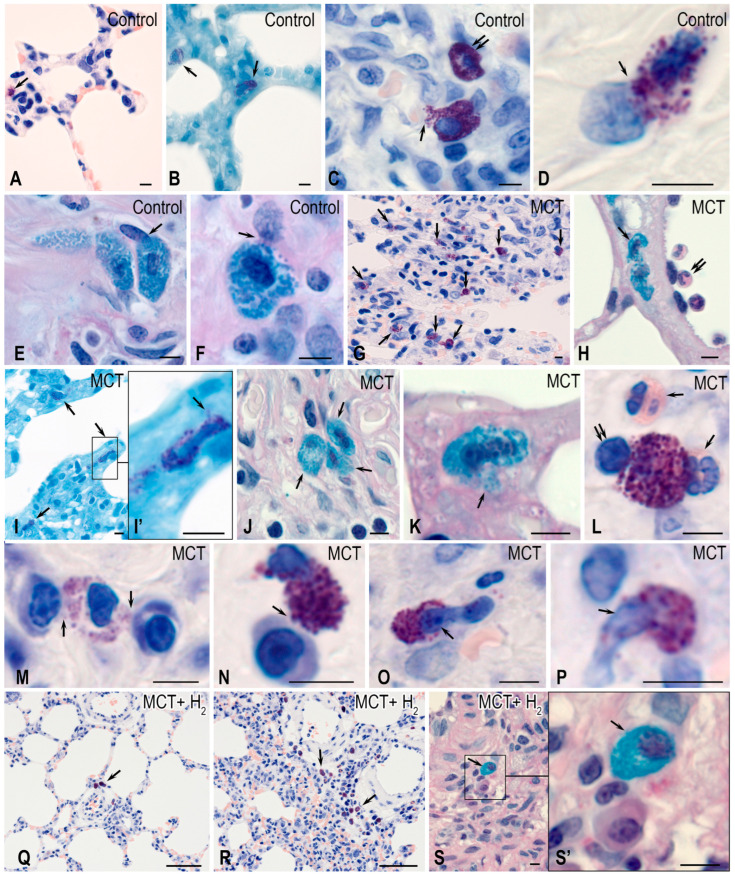
The histochemical features of mast cells (MCs) detected in the lungs of rats in the experiment. Techniques: (**A**,**C**,**D**,**G**,**L**–**R**) Giemsa stain; (**B**,**I**) brilliant cresyl blue staining; (**E**,**F**,**H,J**,**K**,**S**) simultaneous staining with Alcian blue and PAS reaction. Notes: (**A**–**F**) The control group. (**A**,**B**) A small number of MCs in the structures of the acini of the respiratory part of the lungs (indicated by arrows). (**C**) The entry of the MC secretome to the basement membrane of the capillary endothelium in the bronchial wall (indicated by arrows) and MCs without signs of degranulation (indicated by double arrows). (**D**–**F**) Various options for interaction with representatives of fibroblast differon in the walls of the airways (indicated by arrows). (**G**–**P**) The MCT group. (**G**) The high content of MCs in the respiratory structures of the lungs (indicated by arrows). (**H**) The migration of MCs in the wall of the acinus (indicated by arrows); neutrophils are colocalized with the endothelium of the blood vessel (indicated by double arrows). (**I**) MCs with active secretion in the wall of the respiratory bronchioles (indicated by arrows). (**I’**) Enlarged fragment (**I**). (**J**) A group of MCs in the adventitia of the bronchus (indicated by arrows). (**K**) Secreting MCs in the wall of the alveoli (indicated by arrows). (**L**) The interaction of MCs with neutrophils (indicated by arrows) and lymphocytes (indicated by double arrows). (**M**,**N**) The variants of MC interaction with plasma cells (indicated by arrows). (**O**,**P**) MCs with signs of denucleation (indicated by arrows). (**Q**–**S**) The group with H_2_ exposure. (**Q**) A low content of MCs, which are localized in the vascular stroma (indicated by arrows). (**R**) The areas of the lungs that retain signs of inflammation and abundant mast cell infiltration (indicated by arrows). (**S**) MC in the adventitia of a large blood vessel (indicated by arrows). (**S’**) Enlarged fragment (**S**). Scale: (**Q**,**R**)—50 μm; others—5 μm.

**Figure 2:**
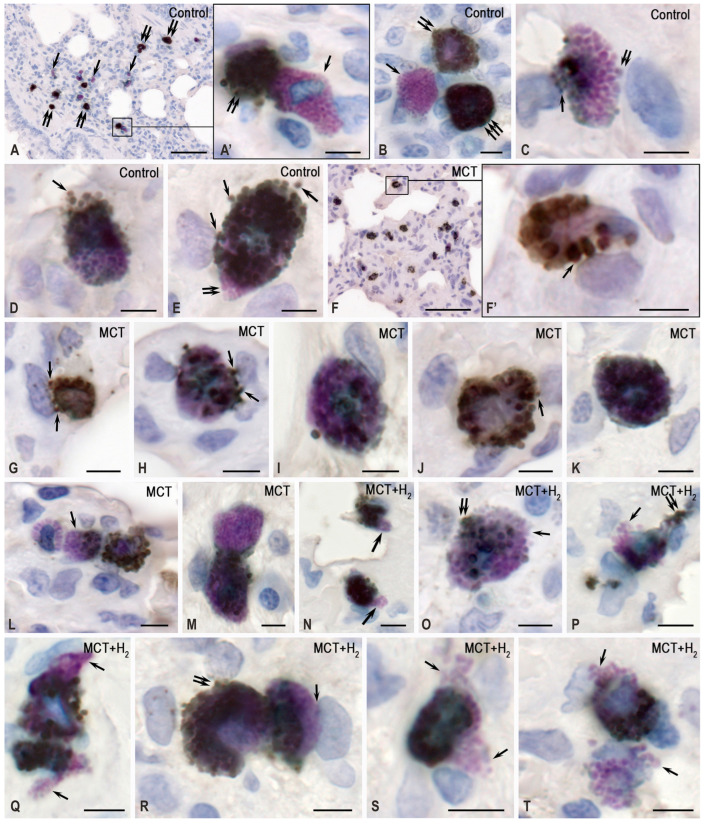
The cytotopographical features of tryptase and the histotopography of mast cells (MCs) in the lungs of rats. Technique: the combined detection of MCs via toluidine blue and immunohistochemical tryptase staining. Notes: (**A**–**E**) the control group, (**F**–**M**) the MCT group, (**N**–**T**) the group with H_2_ exposure. (**A**) MCs with tryptase (indicated by double arrows) and without tryptase (indicated by arrows). (**A’**) A magnified fragment of (**A**). (**B**) MCs without tryptase (indicated by arrows) and with moderate (indicated by double arrows) and high (indicated by triple arrows) tryptase content. (**C**) The differential secretion of tryptase (indicated by arrows) and heparin (indicated by double arrows). (**D**,**E**) The secretion of tryptase-positive granules to targets in the tissue microenvironment (indicated by arrows). (**F**) The presence of tryptase in almost all MCs. (**F’**) A magnified fragment of (**F**), with the selective secretion of tryptase to the fibroblast karyolemma (indicated by arrows). (**G**–**K**) The entry of tryptase-positive granules to the nuclei of neighboring cells in the tissue microenvironment (indicated by arrows). (**L**–**M**) The intercellular exchange of tryptase by neighboring MCs (indicated by arrows). (**N**–**T**) The selective secretion of tryptase-positive (indicated by double arrows) granules and increased supply of tryptase-negative granules (indicated by arrows) to targets in the tissue microenvironment. Scale: (**A**,**F**)—50 μm, others—5 μm.

**Figure 3:**
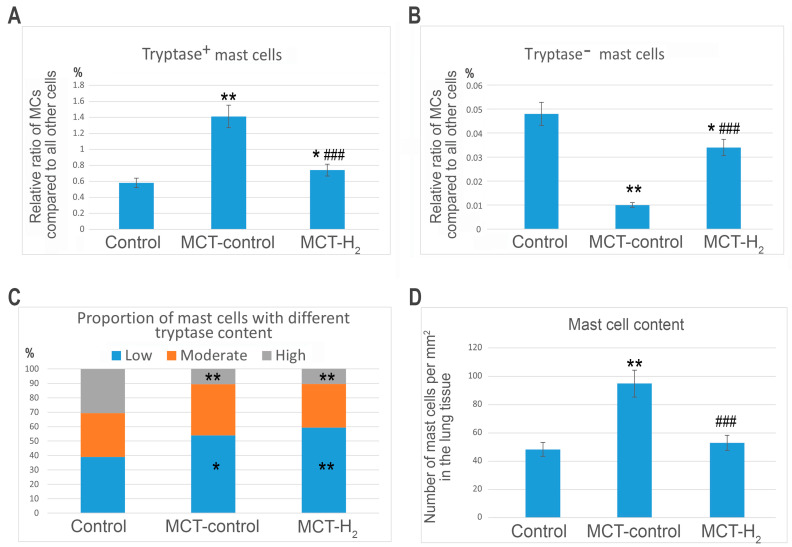
Molecular hydrogen (H_2_) suppressed the MCT-increased number of tryptase-positive mast cells (MCs) while canceling the MCT-decreased number of tryptase-negative MCs. Notes: (**A**) The relative content of tryptase-positive MCs (%) in relation to the total amount of all cell populations in the lungs (in the section). (**B**) The relative content of tryptase-negative MCs (%) in relation to the total amount of all cell populations in the lungs (in the section). (**C**) The relative ratio of the different content of tryptase (low, moderate, and high) in MCs (%). (**D**) The number of tryptase-positive MCs per mm^2^ of the tissue. * *p* < 0.05 compared to the control, ** *p* < 0.01 compared to the control, ### *p* < 0.01 compared to the MCT control.

In the lungs of rats from the control group, MCs were distributed predominantly in the stroma of the airways. They constituted less than 1% of the total cell populations of the organ (Figure 1, Figure 2 and Figure 3). MCs were rarely found in the respiratory part of the lungs, accompanying elements of the vascular bed. The walls of the alveoli and respiratory bronchioles contained the smallest MCs, smaller than 7 μm, with fine metachromatic granularity when stained using various histochemical techniques (Figure 1A,B). Immunohistochemical analysis demonstrated that most lung MCs were tryptase-positive (Figure 2A–E and Figure 3A). The MCs in the adventitia of the bronchi and vascular bed were the largest, with a considerable number of sectoral granules that filled the cytoplasm (Figure 1C–F). 

**Figure 4:**
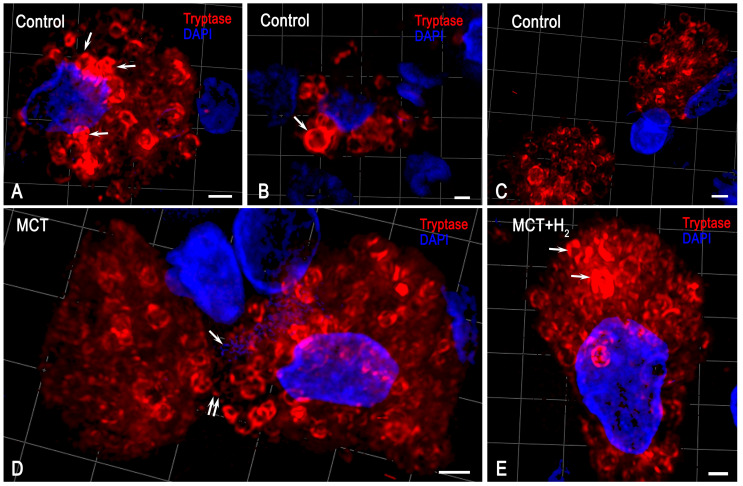
The spatial phenotyping of tryptase in rat lung mast cells (MCs). Technique: immunohistochemical tryptase staining. Three-dimensional models of intracellular localization of tryptase are indicated in Supplements S1–S4. Notes: (**A**–**C**) The control group. (**A**,**B**). Large tryptase-positive granules are located predominantly in the perinuclear region (indicated by an arrow). The peripheral localization of tryptase in secretory granules. (**C**) MCs are located at a paracrine distance from each other. (**D**) The MCT group. Secretory granules decrease in size, and the peripheral intragranular location of trypase is maintained. The contact of MCs with each other is indicated by a double arrow. The extracellular structures produced by MCs (MCETs) are similar to the extracellular traps described for neutrophils (NETs) [46] (indicated by an arrow). (**E**) The group with H_2_ exposure. A lower number of secretory granules with high tryptase content, distant from the nucleus (arrow). The absence of MCETs. Scale: 1 μm.

Secretory processes in the MCs exhibited low intensity, primarily involving the transfer of granules into the extracellular matrix (Figure 1C,D). Furthermore, metachromatic MCs lacking tryptase were detected (Figure 2A–E and Figure 3B). Notably, the protease content in the MCs displayed variability, with some having low levels and others high (Figure 2B and Figure 4A–C). MCs with a low level of protease contained small, limited tryptase-positive granules in the cytoplasm. In contrast, more than 1/3 of the MCs were completely filled with granules with preformed mediators, including tryptase (Figure 2A,D,E and Figure 3C). The MCs displayed moderate secretory activity, frequently demonstrating selective tryptase secretion to various cellular and extracellular targets within the tissue microenvironment (Figure 2A’–E and Figure 4A–C, Supplements S1 and S2). 

Subcutaneous administration of monocrotaline (MCT) led to the development of inflammatory changes in the lung parenchyma, causing reliable changes in all studied MC parameters. Firstly, an increase in intrapulmonary MCs was detected (Figure 1G, Figure 2F and Figure 3A). This was revealed when calculating per mm^2^ of tissue and determining the relative content of MCs compared with other cells of the parenchyma and stroma of the lungs of experimental animals (Figure 3D). An increase in MCs of 143% compared to the control group was accompanied by active secretion. This resulted in an increased proportion of MCs with a low tryptase level in the cytoplasm (Figure 3A). In addition, the MC histotopography, as well as the objects of targeted secretion of tryptase and other components of the secretome, changed (Figure 1H–N and Figure 2F–M). MCs accumulate in certain loci of both the pneumatic and respiratory sections of the lungs, sometimes creating extensive intraorgan subpopulations (Figure 1G,H–N, Figure 2F and Figure 4D, Supplement S3). 

MCs within these histological compartments were in active contact with other cells within the lung tissue microenvironment. Notably, there was evidence of secretory interaction with cells of the fibroblast phenotype (Figure 2F’,G–J). In several instances, it appeared that tryptase-positive granules were in direct contact with the nuclear membranes of adjacent cells. In such cases, the size of the granules varied, ranging from larger (surpassing 1 μm) to smaller dimensions (Figure 2F’,G–H).

In this way, epigenetic regulation of MCs of other lung cells could be realized through the transfer of tryptase into the nucleoplasm and further interaction with histones, resulting in altered functions [44,45]. In addition, tryptase-positive components of the secretome could be seen in multiple nuclei (Figure 2G–H).

Exposure to MCT resulted in the development of large granules, some exceeding 1 μm or larger, within many MCs. These granules were dispersed throughout the cytoplasm or in direct contact with the MC’s plasma membrane, which enabled the transgranulatation of tryptase to targets in the local tissue microenvironment (Figure 2F). Indeed, in terms of cytotopography, in MCs, there was a redistribution of tryptase-containing secretory granules from the peripheral regions of the cytoplasm to the periplasmalemmal regions. 

MCs frequently contacted plasma cells, and the directed secretion process led to the translocation of tryptase into the nuclei of plasma cells, forming tryptase-positive loci. MC contacts with neutrophilic granulocytes were frequently observed, and in some cases, MCs interacted with several immunocompetent cells, either granulocytes or agranulocytes (Figure 1L–N). Small MCs were discovered in the respiratory section, and it seemed that their nuclei were released from the cytoplasm containing secretory granules. This phenomenon could be regarded as a reoccurring pattern (Figure 1O,P). Moreover, in some cases, most of the nucleus was outside the granule-containing cytoplasm of the MC (Figure 1O,P).

Importantly, areas with MCs and tryptase accumulations within the extracellular matrix fostered the development of niches with a profibrogenic phenotype. These niches initiated a program of fibrotic morphogenetic transformations of the local tissue microenvironment upon exposure to MCT. The intercellular exchange of tryptase in MCs was activated; in these conditions, tryptase was evidently able to move from one cell to the neighboring one and then to others, realizing certain signal-inducing effects on the regulation of the functional phenotype (Figure 2L,M).

In particular, such signals could serve as a stimulus for the onset of tryptase expression in MCs that had not previously synthesized the protease (Figure 2L,M). 

The number of metachromatic MCs without tryptase was significantly reduced compared to animals from the biological control group (Figure 2L,M and Figure 3B). In some MCs, a certain “polarization” with a predominant accumulation of tryptase in one of the cytoplasmic compartments was detected (Figure 2L). 

Following MCT exposure, the content of large connective tissue MCs, previously located primarily in the stroma of large airways or intrapulmonary vessels, increased in the walls of acini and respiratory bronchioles. The secretory activity of the mucous cells of the airway epithelium exhibited such heightened secretory activity that all of the goblet cells within the ciliated epithelium of the mucous membrane appeared depleted.

The inhalation of H_2_ had several effects on the state of the local tissue microenvironment of the lungs, affecting both the cellular component and the structures of the extracellular matrix. Overall, using H_2_ reduced the inflammatory changes in the lungs induced by MCT, which correlated with the suppression of the proinflammatory effect of the intraorgan MC population. First, H_2_ exposure led to a decreased size of the MC population in the acini and other structures of the lungs compared to similar parameters in the MCT group (Figure 3A,D). MCs were localized predominantly in the stroma of the airways, and MC accumulations in the respiratory part of the lungs decreased (Figure 1Q–S). Although inflammatory lesions remained in the lungs, their number was reduced compared to parameters detected in animals exposed to the MCT effect alone (Figure 1Q–S). 

The content of tryptase per mast cell remained lower than the similar values in the control group (Figure 3C, Supplement S4). However, in some MCs, there was an impression that they were overfilled with tryptase-containing granules (Figure 4E). Moreover, secretion in numerous MCs was accompanied by the excretion of metachromatic granules without tryptase (Figure 2N–T). 

These facts indicate certain effects of molecular hydrogen in blocking the release of a specific protease into the extracellular matrix of the local tissue microenvironment of the lungs. Concurrently, there was increased heparin excretion, as evidenced by metachromasia of the secreted granules (Figure 2P,S,T). In the broader population of MCs within the lungs, the number of metachromatic MCs without tryptase increased compared to the MCT group, though it did not approach the level observed in the control group (Figure 3B). Furthermore, there was an observed shift in the selectivity of the qualitative composition of mediators during the targeted secretion of MCs in relation to fibroblastic differential cells. For example, patterns indicated the secretion of heparin-containing granules devoid of tryptase toward fibroblasts and fibrocytes.

**Figure 5:**
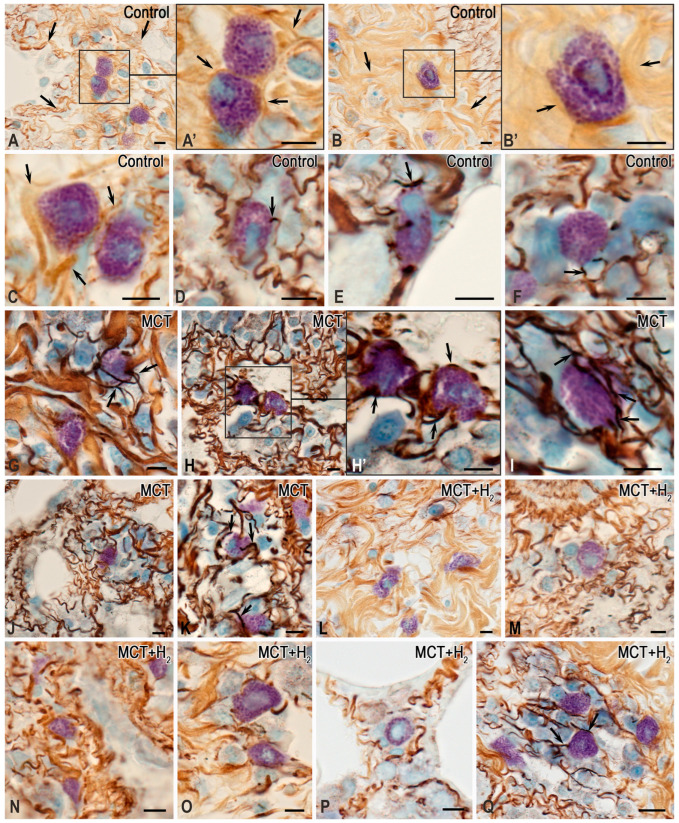
Mast cells (MCs) in the MCT-induced remodeling of the collagen extracellular matrix in the lungs of rats. Technique: combined staining with toluidine blue and silver impregnation. Notes: (**A**–**F**) the control group; (**G**–**K**) the MCT group; (**L**–**Q**) the group of H_2_ exposure. (**A**–**C**) MCs in the structures of large bronchi, located among bundles of mature golden-yellow collagen fibers (indicated by arrows). (**A’**) Enlarged fragment (**A**). (**B’**) Enlarged fragment (**B**). (**D**–**F**) MCs are colocalized with reticular fibers (indicated by arrows) in the stroma of the respiratory part of the lungs. (**G**) The impregnated fibers in the stroma of a large bronchus colocalized with a mast cell (indicated by arrows). (**H**–**K**) Reticular fibers in the stroma of the respiratory part of the lungs; many are adjacent to the MCs (indicated by arrows). (**H’**) Enlarged fragment (**H**). (**L**–**Q**) The low number of reticular fibers in the lungs with preservation of fibrillogenesis foci in some loci of the tissue microenvironment ((**Q**), indicated by arrows). Scale: 5 μm.

**Figure 6:**
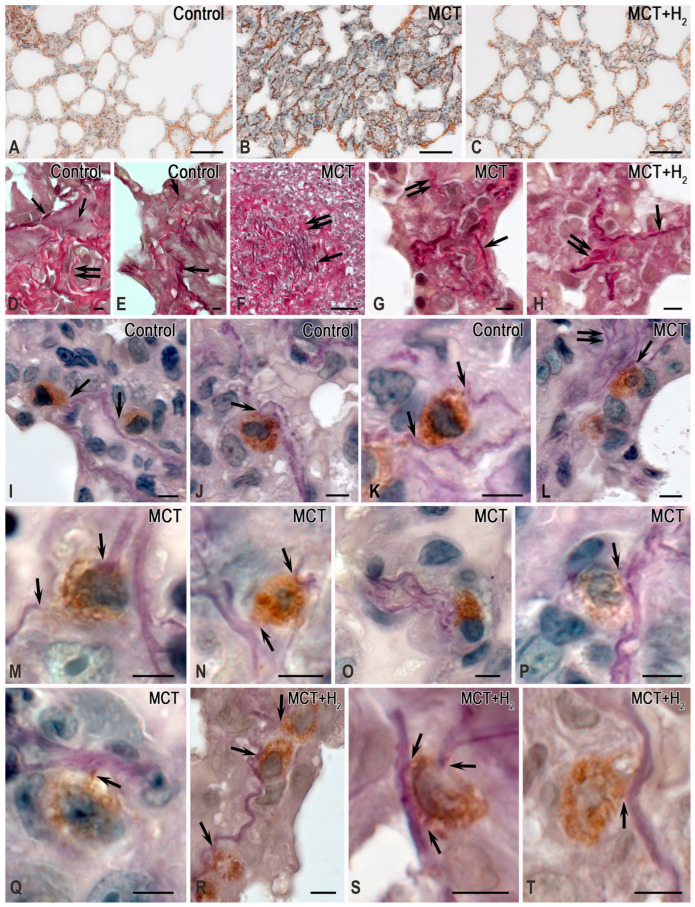
The effects of H_2_ on the remodeling of elastic and collagen fibers in the stroma of the lungs of rats with MC participation. Techniques: (**A**–**C**) Combined toluidine blue and silver impregnation staining. (**D**–**H**) Combined Weigert and van Gieson staining. (**I**–**T**) Combined staining of elastic fibers according to Weigert (dark violet) and the immunohistochemical detection of MC tryptase (brown). (**A**,**D**–**E**,**I**–**K**) The control group. (**B**,**F**–**G**,**L**–**Q**) The MCT group. (**C**,**H**,**R**–**T**) The group with H_2_ exposure. Notes: (**A**–**C**) Low (**A**,**C**) and high (**B**) levels of collagen fibrillogenesis in the lungs. (**D**–**H**) The fibrous stroma of the lungs. The presence of collagen (indicated by double arrows) and elastic (indicated by arrows) fibers is predominantly in the structural membranes of the airways. MCT causes an increase in lung fibers (**F**,**G**), which significantly decreases when exposed to H_2_ (**H**). (**I**–**K**) Predominant MC colocalization with elastic fibers in the vascular bed or airways (indicated by arrows). (**L**–**Q**) An increase in the content of elastic fibers in the local tissue microenvironment of the respiratory part of the lungs after MCT exposure, with frequent contact with MCs (indicated by arrows). (**R**–**T**) MCs adjacent to elastic fibers after MCT exposure combined with H_2_ exposure (through an inhaled respiratory mixture). Scale: (**A**–**C**): 50 μm; others: 5 μm.

The lung stroma in the control group was characterized by a prevalence of collagen fibers over the elastic component, with reticular fibers representing over half of the total fibrous component area (Figure 5A–F). In comparison, the respiratory portion of the lung exhibited a less pronounced fibrous component, especially when contrasted with the well-defined bundles of collagen and elastic fibers located between the membranes of large bronchi and intraorgan blood vessels. Simultaneously, mature collagen fibers dominated the adventitia of the airways and large vessels (Figure 5A–C and Figure 6A). MCs in the lungs of intact animals demonstrated minimal involvement in stromal remodeling. 

MCs predominantly occupied spaces amid mature collagen fibers, which were part of the airway structures, and did not exhibit activity in collagen fibrillogenesis. In this case, the fibrous component was represented predominantly by mature collagen fibers with a high content of type I collagen (Figure 5A–F). MCs were located between thick bundles of collagen fibers (Figure 5A–C). Much less frequently, MCs were detected in the respiratory region in contact with impregnated reticular fibers with a high content of type III collagen (Figure 5D–F). 

**Figure 7:**
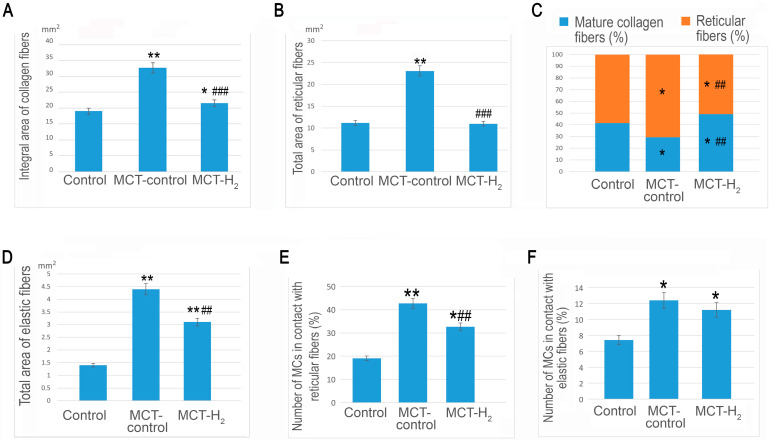
H_2_ suppressed the MCT-increased collagen, reticular, and elastic fibers, as well as the number of mast cells (MCs) contacting with reticular fibers. (**A**) The total area of collagen fibers in the connective tissue of the lungs. (**B**) The general content of reticular collagen fibers in the lungs. (**C**) The collagen fiber composition profile in the interstitium of the lungs. (**D**) The total area of elastic fibers in the structural components of the lungs. (**E**) The assessment of the frequency of mast cell contact with reticular fibers in lung connective tissue. (**F**) An analysis of the colocalization of MCs with elastic fibers in the lungs. All lung tissues were taken from the area without the lumen of the airways, alveoli, and vascular bed. * *p* < 0.05 compared to the control, ** *p* < 0.01 compared to the control, ## *p* < 0.05 compared to the MCT control, ### *p* < 0.01 compared to the MCT control.

MCT exposure increased the content of elastic fibers in the lungs, which was observed both in the stroma of large airways or vessels and the interalveolar septa (Figure 6F,G and Figure 7D). Concurrently, there was an increase in the content of collagen fibers due to a significant increase in reticular fibers (Figure 5G–K, Figure 6B and Figure 7A–C). 

The evaluation of the compositional profile of the collagen fibrous component in the lungs demonstrated an increased content of reticular fibers, which became more numerous both in the respiratory part of the lungs (Figure 5H–K) and in the stroma of the airways (Figure 5G). A crucial effect induced by MCT was a considerable increase in the number of elastic fibers within the lung stroma compared to the control group (Figure 6F,G,M–P). This shift in the spatial distribution of MCs within the local tissue microenvironment, coupled with active involvement in collagen fibrillogenesis, is particularly noteworthy (Figure 5G–K). In fact, MCs surrounded by impregnated fibrous structures were detected in every area of the local tissue microenvironment. Such patterns were rarely detected in the control group (Figure 5A–F). In contrast, in the MCT group, even in the initial visualization, predominant MC colocalization with fine and medium-caliber reticular fibers was detected (Figure 5G–K). It should also be noted that MCs were more frequently colocalized with elastic fibers (Figure 6L–P). 

The effects of H_2_ were also detected in the phenotype of the extracellular matrix of the lung stroma. Thus, compared to animals exposed only to MCT, the integral area of collagen fibers significantly decreased in rats exposed to H_2_. Nevertheless, it did not approach the level of animals from the control group in relative values (Figure 7A). The decreases in the content of the fibrous collagen component occurred mainly due to reticular fibers (Figure 7B). 

When determining the collagen fibrous component, the number of reticular fibers decreased compared to the level of the MCT exposure group, reaching the level of the control group (Figure 7B). An analysis of the elastic fiber areas demonstrated that, although H_2_ exposure did not result in the same level as the control group, it reduced the formation of the elastic network in the lungs compared to the MCT exposure group. Concurrently, examination of the profiles of collagen and elastic fibers revealed that H_2_ exposure led to an increase in the relative proportion of the elastic component in the lungs compared with the parameters of animals from the control group and rats from the MCT group. Thus, H_2_ exposure caused a decrease in MC colocalization, with reticular fibers having histochemical signs of collagen fibrillogenesis both in the stroma of the pneumatic and respiratory sections of the lungs (Figure 7E). The complete elimination of the consequences of the toxic effects of MCT was not achieved, as extensive compartments with signs of active collagen fibrillogenesis with MC participation continued to be found in the lungs (Figure 5Q).

**Figure 8:**
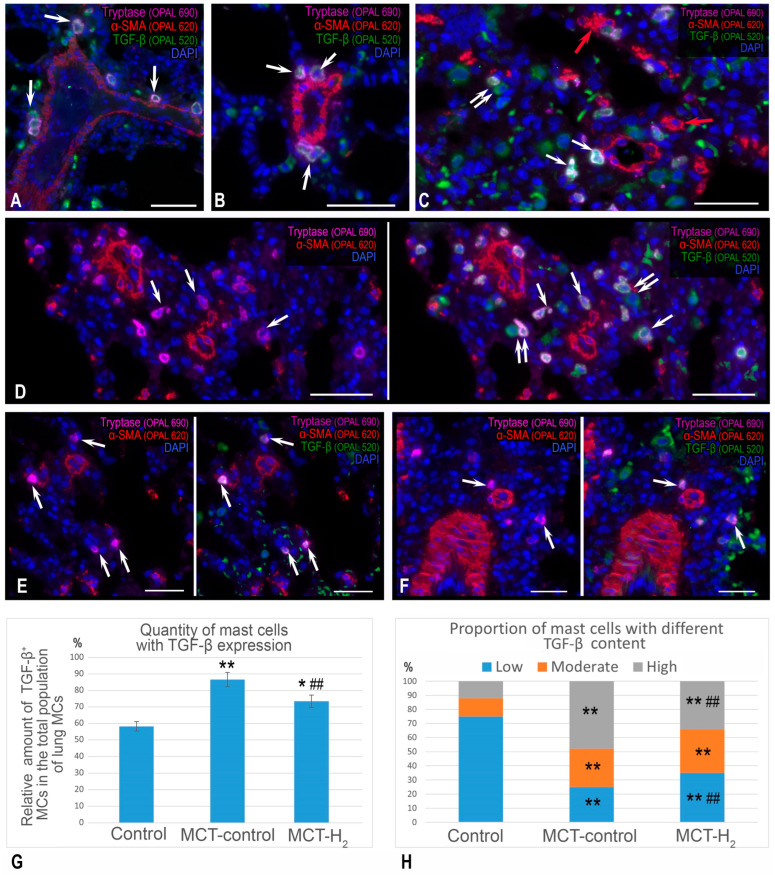
The effects of H_2_ on the features of TGF-β expression in MCs in the lungs of rats. Techniques: multiplex immunolabeling of tryptase, TGF-β, and α-SMA. Notes: (**A**,**B**) The control group. The predominant location of tryptase-positive MCs with moderate expression of TGF-β in the stroma of the bronchial tree and the adventitia of large vessels (indicated by arrows). (**C**,**D**) The MCT group. An increased number of MCs in the respiratory part of the lungs with a high expression of TGF-β (indicated by arrows) and increased frequency of colocalization with other TGF-β-positive cells (indicated by double arrows). Myofibroblasts (presumably, indicated by red arrows) were detected (**E**,**F**). The group with H2 exposure. A decreased number of MCs (indicated by arrows) and TGF-positive cells in the lungs. (**G**) The TGF-β expression profile in the mast cell population. (**H**) The ratio of MCs with different TGF-β contents (%). * *p* < 0.05 compared to the control, ** *p* < 0.01 compared to the control, ## *p* < 0.05 compared to the MCT control. Scale: 50 μm.

In the control group, TGF-β expression was detected in most MCs of the lungs and was characterized by predominantly low levels (Figure 8A,B,G,H). After MCT exposure, the content of TGF-β, which was identified in all MCs, increased (Figure 8C,D,G,H). MCs were more often colocalized with other TGF-β-positive cells of the respiratory and pneumatic parts of the lungs. Consequently, they had an inducing effect on the formation of the fibrogenic phenotype of a specific tissue microenvironment.

When exposed to MCT, cells expressing α-smooth muscle actin (α-SMA) were detected that were not part of the structural components of the airways or vascular bed (Figure 8C), which could indicate increased activity of fibroblast-to-myofibroblast transformation. Using molecular hydrogen led to a reduction in the count of TGF-β positive cells within the lungs, accompanied by a decrease in TGF-β expression in MCs (Figure 8E,F,G,H). Compared to animals from the control group, there were persistent lesions with evident signs of inflammation and accumulations of immunocompetent cells in the lungs of rats. Although the severity of such histopathological patterns varied significantly in rats from experimental groups, in general, infiltration of immunocompetent cells in various parts of the lungs was smaller compared to animals only exposed to MCT. In addition, practically no contacts of MCs with neutrophils were detected.

## 3. Discussion

One prevalent perspective supporting the multiple positive effects of H_2_ posits that H_2_ reduces oxidative-stress-related damages and curtails the intensity of inflammatory cytokine secretion [26,27,47]. 

There is evidence that the beneficial effects of molecular hydrogen may be associated with the activation of the nuclear factor erythroid 2-related factor 2 pathway, which promotes innate antioxidants and a reduction in apoptosis, as well as inflammation [48]. H2 has been demonstrated to suppress NF-κB inflammatory signaling and induce the Nrf2/Keap1 antioxidant pathway, as well as to improve mitochondrial condition and enhance cellular bioenergetics [49]. H_2_ activates the system, which manages cell redox balance, fortifying antioxidant defenses and scavenging ROS to counteract oxidative stress [50]. H_2_ may be superior to conventional antioxidants since it can selectively reduce ●OH radicals while preserving important ROS otherwise used for normal cellular signaling. H_2_ influences the crosstalk among the regulatory mechanisms of autophagy, which involve p53, MAPKs, p38 MAPK, NF-κB, Nrf2, mTOR, and so forth [51]. H_2_ protected mitochondrial morphology and function in melanocytes under stress and promoted the activation of Nrf2 signaling [52]. Molecular hydrogen reduces ischemia or reperfusion injury via different mechanisms. It has been shown in different models, including heart failure, cerebral stroke, transplantation, and surgical interventions [53]. Despite such a significant amount of molecular data on the possibility of developing protective properties of H_2_ against the effects of free radicals, histological data on its antioxidant effects at the level of structural components of tissue microenvironment are still lacking. As the main target of our study, we chose MCs as the most important component for maintaining the local homeostasis of both the immune and stromal landscape. MCs are one of the crucial components of the immune landscape of the specific tissue microenvironment of any organ, including the lungs. Possessing multiple effects, MCs have the potential to provide diverse regulatory effects related to various pathological processes [10,15].

In particular, MCs are key players in developing the proinflammatory local tissue microenvironment, acting as conductors of intercellular signaling to attract and regulate the functional activity of other immunocompetent cells [16,20,21,54,55]. This allows MCs to be the most important inducers, providing the formation of fibrogenic niches in organs and conductors and providing the development of a persistent fibrotic phenotype of the local tissue microenvironment [41,56,57,58].

In our study, we identified the modifying effects of H_2_ on MCT-induced fibrotic phenotype formation in the local tissue microenvironment of the lungs. Indeed, the specific protective effects of H_2_ against the proinflammatory effect of MCT included decreased formation of the fibrous extracellular matrix in the lung stroma. In particular, MCT-induced inflammatory changes in the respiratory regions of the lungs, as well as the formation of reticular fibers and the severity of the profibrogenic phenotype of the local tissue microenvironment, were decreased. 

The migration of the connective tissue MCs with a high content of heparin and other polyanions to certain loci of the respiratory part of the lungs supports their participation in directly inducing effects on the assembly of collagen fibers from microfibrils. Undoubtedly, to start the polymerization of fibrous collagen proteins (types I and III) into supramolecular fibrous structures, a strict ratio of the components of the local tissue microenvironment is required. These include water, proteoglycans, ATP, salts, and other substances that provide favorable conditions for critical monomer convergence of collagen proteins and dimer formation [59].

In particular, it is compulsory to reduce the concentration of water molecules that prevent the combination of collagen monomers into dimers. Heparin secretion provided by MCs promotes fibrillogenesis, as this glycosaminoglycan reduces the content of water molecules in the local volume of the tissue microenvironment, thus promoting the formation of collagen dimers [59]. The secretion of chymase, located in the connective tissue, enzymatically performs proteolysis of type I procollagen into type I collagen. Consequently, this also accelerates the accumulation of collagen monomers prepared for polymerization in the local tissue microenvironment. The collagen molecule acquires one large central triple helical domain and terminal short non-collagenous sequences called telopeptides because of complete processing and becomes capable of further self-organization into a microfibril [60]. Collagen nucleation sites are formed in the periplasmal membrane areas of fibroblasts or myofibroblasts. At these sites, macromolecular complexes of fibril-forming collagen are sequentially assembled from monomeric subunits with further linear and lateral growth of fibers [61]. The first steps of collagen maturation outside the cytoplasm of fibroblasts or myofibroblasts for fibrillogenesis include the cleavage of amino- and carboxy-terminal pro-peptides and the formation of tropocollagen involving several enzymes [60,62]. The enzymes that process a particular pro-peptide are specific for each type of collagen. Tropocollagen molecules can be a guide in the formation of collagen fibrils. The growth of the latter requires collagens of types I, III, and V, as well as non-fibrillar collagen molecules necessary to arrange interaction with other structural elements of the intercellular matrix [63] and small proteoglycans (decorin, biglycan, fibromodulin, etc.) [59]. Notably, type V collagen determines the processes of fibril thickening [64,65]. Thus, MCs can be directly involved in inducing collagen fiber self-organization in the intercellular matrix of fibrogenic niches or local tissue microenvironment with a profibrogenic phenotype. In this respect, it is critical to consider MCs’ interaction with fibroblasts and fibrocytes.

Using H_2_ as a substance with antioxidant properties represents the most pivotal mechanism in reducing the secretory activity of MCs, thereby holding a theoretically promising potential to reduce the inflammatory specific tissue microenvironment. Our results suggested that MCs are effective targets for H_2_ in the local tissue microenvironment [36,39]. In our experiment, we found that the positive dynamics of fibrotic changes developing in the lungs correlated with the MC phenotype. It should be noted that H_2_ exposure initially results in a decreased level of tryptase in the lungs, which has pronounced proinflammatory effects [66,67]. A reduced volume of the tryptase-positive mast cell population, as well as the possible blockade of tryptase secretion into the extracellular matrix, will help reduce inflammation as well as fibrotic changes, as MC tryptase is known to stimulate the synthesis of type I collagen in human lung fibroblasts [68]. Accordingly, H_2_ can reduce the formation of a fibrotic phenotype in the local tissue microenvironment of the lungs, which is a direct continuation of inflammatory phenomena due to MCT exposure. There are also direct and indirect tryptase effects in relation to the intensity of collagen fibrillogenesis, in particular, stimulation of the migration, division, and biogenesis activity of fibrous collagens in fibroblasts. Moreover, tryptase promotes the transformation of fibroblasts into myofibroblasts, which has significantly greater activity in forming extracellular matrix components [69,70]. Based upon the activity of **α**-SMA expression in lung cells, which are not related to the smooth muscles of the airways and vascular bed, and the number of lung cells, MCT exposure results in an increased number of myofibroblasts in the stromal landscape of the lungs.

Thus, the data obtained in this experiment suggest certain morphological criteria for elucidating the anti-inflammatory effects of H_2_ involving MCs. These novel mechanisms through which H_2_ induces biological effects will help validate personalized approaches for its application in translational medicine.

## 4. Materials and Methods

### 4.1. Experimental Design

The experiments were conducted on 2-month-old male Wistar rats (180–220 g b.w.) according to our previous report [7]. All of the manipulations with the animals were conducted according to the Council Directive 86/609/EEC principles. The animals were obtained from the vivarium of the Research Institute of General Pathology and Pathophysiology (Moscow, Russia). The rats were kept under 12 h daylight conditions with free access to water and food. Humidity and temperature control were also performed. The adaptation period after transport was at least 7 days.

On day 1, two groups of animals received a single subcutaneous injection of MCT (60 mg/kg in 60% ethyl alcohol) (Sigma Aldrich, Darmstadt, Germany). The control group received only a solvent for MCT (60% ethyl alcohol) subcutaneously; that is, it was the control for the MCT effect. 

Animals receiving the MCT injection were divided into two groups: those ventilated with room air (MCT control) and those ventilated with a mixture of room air with 4% hydrogen (MCT-H_2_). The ventilation of all cages was continuous except for the period of cage cleaning (1h/3 days). Animals inhaled either air or air containing 4% H_2_ for 21 days. There were four rats in each cage and two cages in each group (control group, n = 8; MCT group, n = 8; and MCT + H_2_ group, n = 8). 

**Figure 9:**
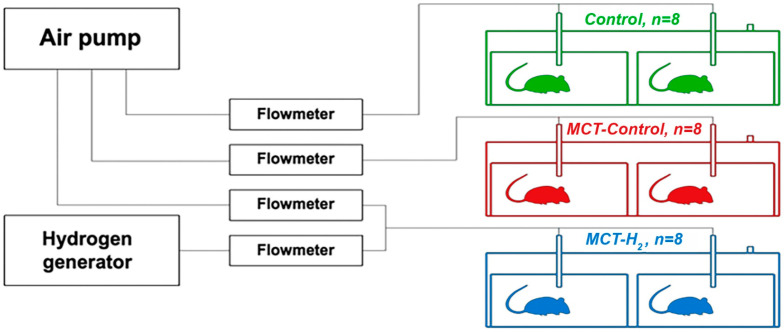
A schematic representation of the experimental setup.

Rats were placed in three 130-liter plastic containers (SAMLA 203.764.41, Inter IKEA Systems, Delft, Netherlands). Two T2 cages for laboratory animals were placed inside each container. To ventilate the containers, a Linear Air Pump (SPP-15GAS, Hiblow XP 40, Techno Takatsuki, Takatsuki, Osaka, Japan) was used. Molecular hydrogen was produced by the generator (Pioneer, OOO, Vodorodpomogaet, Moscow, Russia). Figure 9 shows a schematic of the experimental setup.

The actual hydrogen and air flow rates were adjusted by the flowmeter rotameters (LZB-3, LZM-4T, Yuyao, Ningbo, China) and kept at 0.15 L/min and 4 L/min, respectively. It created 3.5–4.0% of the hydrogen in the air, which was used for c ventilation of the cage with the MCT-H2 group of rats. The real concentration of H_2_ in the air of the MCT-H2 group’s ventilated cage was measured periodically using gas chromatography (TRIlyzer mBA-3000, Taiyo Instruments, Osaka, Japan). At the end of the experiments, euthanasia was performed on urethane-narcotized rats via decapitation.

### 4.2. Histoprocessing 

After 24–48 hours of fixation in 10% neutral formalin at room temperature, the lungs of rats were embedded in paraffin during a standard sample-preparation procedure in a Leica ASP 6025 closed-type automatic histoprocessor. To conduct histochemical and immunohistochemical analysis using a fully motorized HistoCore AUTOCUT rotary microtome (manufactured by Leica Biosystems, Nussloch, Germany), total histological sections of the left lung, 5 and 2 μm thick, respectively, were prepared.

### 4.3. Tissue Probe Staining

For the immunohistochemical assay, we subjected deparaffinized sections to antigen retrieval by heating the sections with R-UNIVERSAL Epitope Recovery Buffer at 95 °C × 30 min. Blocking the endogenous Fc receptors prior to incubation with primary antibodies was omitted, according to our earlier recommendations [71]. The list of primary antibodies used in this study is presented in Table 1. 

Immunohistochemical visualization of bound primary antibodies was performed manually, according to the standard protocol [72,73]. For manually performed immunostaining, primary antibodies were incubated overnight at +4 °C. Bound primary antibodies were visualized using secondary antibodies conjugated with Alexa Fluor-488 or Cy3. Single and multiple immunofluorescence labeling were performed according to standard protocols [72]. The list of secondary antibodies and other reagents used in this study is presented in Table 2.

Sequential multiplex immunohistochemical staining for the simultaneous detection of tryptase, TGF-β, and α-SMA was performed in accordance with Akoya Biosciences (Marlborough, MA, USA) recommendations on the use of OPAL series fluorochromes for the Mantra 2 Quantitative Pathology Imaging System. In addition, when using OPAL series fluorochromes for repeated retrieval, the EZ—Retriever^®^ System, MW015-IR (BioGenex, Fremont, CA, USA), was applied. 

For the simultaneous detection of tryptase-positive MCs, elastic fibers, and collagen fibers, a combined protocol, including standard immunohistochemical tryptase staining (Table 1) and elastic fiber staining according to Weigert’s method (Table 2) was used, or a protocol for simultaneous staining of elastic and collagen fibers according to the combined Weigert–Van Gieson method (Table 2) [74]. The ratio of elastic and collagen fibers was estimated according to the manufacturer’s protocol after their simultaneous Weigert–Van Gieson staining (Table 2). 

A combined approach for assessing tryptase expression in the mast cell population was performed according to the original two-step immunohistochemical detection method of tryptase and toluidine blue staining [75]. 

Histochemical staining with Giemsa solution, hematoxylin, silver impregnation, and combined Weigert–Van Gieson staining was performed according to the manufacturer’s instructions (Table 2). 

Control incubations included the omission of primary antibodies or the substitution of primary antibodies by the same IgG species (Dianova, Hamburg, Germany) at the same final concentration as the primary antibodies. The exclusion of either the primary or the secondary antibody from the immunohistochemical reaction and the substitution of primary antibodies with the corresponding IgG at the same final concentration resulted in a lack of immunostaining. The specific and selective staining of different cells with the use of primary antibodies from the same species on the same preparation is, by itself, a sufficient control for the immunostaining specificity.

### 4.4. Quantitative Analysis

Quantitative analysis of the studied criteria involved total lung sections in the same plane. Planimetric analysis was performed to identify the number of MCs per unit area of the lung, the absolute and relative number of MCs and other cells, the ratio of the elastic and collagen fiber areas, and to specify the profile of mast cell tryptase with the determination of “high”, “moderate”, and “low” expression levels and the intracellular profile of MCs by tryptase content and metachromasia using the QuPath v0.5.1 software product [76]. Stained sections were scanned using the ×40 objective of ScanScope CS (Leica Biosystems, Deer Park, IL, USA), the Mantra 2 Quantitative Pathology Imaging System (Akoya Biosciences, Marlborough, MA, USA) based on Olympus BX43 microscope and a digital pathology slide scanner (fluorescence) KF-FL-005 (Ningbo, ZheJiang, China).

### 4.5. Image Acquisition

Stained tissue sections were observed on a ZEISS Axio Imager.Z2 equipped with a Zeiss alpha Plan-Apochromat objective 100×/1.46 Oil DIC M27, a Zeiss Objective Plan-Apochromat 150×/1.35 Glyc DIC Corr M27, and a ZEISS Axiocam 712 color digital microscope camera and ZEISS Axiocam 712 mono digital microscope camera (Carl Zeiss Vision, Jena, Germany). Captured images were processed with the software program “Zen 3.0 Light Microscopy Software Package”, “ZEN Module Bundle Intellesis & Analysis for Light Microscopy”, “ZEN Module Z Stack Hardware” (Carl Zeiss Vision, Jena, Germany) and submitted with the final revision of the manuscript at 300 DPI. Photomicrographs were obtained in some cases with a Nikon D-Eclipse C1 Si confocal microscope based on Nikon Eclipse 90i. The Mantra 2 Quantitative Pathology Imaging System for multiplex visualization (Akoya Biosciences, Marlborough, MA, USA) based on an Olympus BX43 microscope equipped with a scientific-grade multispectral 12-bit monochrome high-sensitivity CCD camera with a liquid crystal tunable spectral filter was used to determine the profile of tryptase—a positive mast cell—in multiple immunodetections of tryptase, TGF-β, and α-SMA using the OPAL 690, OPAL 520, and OPAL 620 fluorochromes, respectively.

### 4.6. Statistical Analysis

Statistical analysis was performed using the SPSS software package (Version 13.0, IBM, Armonk, New York, United States). The results are presented as the mean (M) ± m (standard error of the mean). To assess the significance of the differences between the two groups, Student’s t-test or the Mann–Whitney U test in the case of a nonparametric distribution was used. Significance was considered as *—*p* < 0.05 compared to the control, **—*p* < 0.01 compared to the control, ##—*p* < 0.05 compared to the MCT control, ###—*p* < 0.01 compared to the MCT control.

## 5. Conclusions

This study is the first to investigate the state of the intrapulmonary MC population under conditions of H_2_ inhalation, including unique histochemical and immunohistochemical staining protocols, to interpret MC-associated mechanisms of extracellular matrix remodeling. The antifibrotic effects of H_2_ on the local tissue microenvironment of the lungs were established. This was mediated by the secretory pathways of MCs in relation to the structural components of the extracellular matrix and other cellular subpopulations. The detected changes in the MC phenotype under simulated monocrotaline pulmonary hypertension with inhaled H_2_ delivery allowed us to conclude that the molecular phenotype, migratory activity, and secretory activity of MCs are sufficiently sensitive to the effects of molecular hydrogen. The decrease in the intraorgan population of tryptase-positive MCs in the lungs, combined with a decreased efficiency of collagen fibrillogenesis, are important factors that contribute to the preventive effects of H_2_ against inflammatory and profibrogenic states of the lungs. MCs as a target of H_2_ is a promising angle for further research, in order to identify the mechanisms responsible for the biological effects of molecular hydrogen in the living body. The data obtained can be used in translational medicine to develop personalized algorithms for H_2_ use to specifically induce anti-inflammatory and anti-fibrotic effects. The limitations of this work are related to the lack of analysis of the interaction of MCs under experimental conditions with other immunocompetent, stromal, and epithelial cells of the lungs, including alveolar type II cells, limiting work on further deciphering the biological effects of H_2_. However, the use of spatial proteomics and multiplex IHC protocols in subsequent studies of the lung tissue microenvironment may shed light on these issues and reveal new mechanisms for the formation of the MC-initiated remodeling effects of H_2_.

## Figures and Tables

**Table 1:** Primary antibodies used in this study.

Antibodies	Host	Catalogue Nr.	Dilution	Source
Tryptase	Mouse monoclonal	#ab2378	1:2500	AbCam, Cambridge, UK
TGF-β	Rabbit monoclonal	#ab215715	1:200	AbCam, Cambridge, UK
Alpha SMA	Mouse monoclonal	#ab124964	1:2500	AbCam, Cambridge, UK

**Table 2:** Secondary antibodies and other reagents.

Antibodies and Other Reagents	Source	Dilution	Label
Goat anti-mouse IgG Ab (#ab97035)	AbCam, Cambridge, UK	1/300	Cy3
Goat anti-rabbit IgG Ab (#ab150077):	AbCam, Cambridge, UK	1/300	Alexa Fluor 488
Secondary antibodies conjugated with horseradish peroxidase (Opal Polymer HRP Ms + Rb (#ARH1001EA))	Akoya Biosciences, Marlborough, MA, USA	ready-to-use	Opal 480 Reagent Pack (#FP1500001KT)
Secondary antibodies conjugated with horseradish peroxidase (Opal Polymer HRP Ms + Rb (#ARH1001EA))	Akoya Biosciences, Marlborough, MA, USA	ready-to-use	Opal 570 Reagent Pack (#FP1488001KT)
Secondary antibodies conjugated with horseradish peroxidase (Opal Polymer HRP Ms + Rb (#ARH1001EA))	Akoya Biosciences, Marlborough, MA, USA	ready-to-use	Opal 690 Reagent Pack (#FP1497001KT)
R-Universal buffer for antigen unmasking/epitope recovery on formalin-fixed, paraffin-embedded sections (#AP0530-500)	Aptum Biologics Ltd., Southampton, SO16 8AD, UK	1:10	*w*/*o*
AmpliStain™ anti-Mouse 1-Step HRP (#AS-M1-HRP)	SDT GmbH, Baesweiler, Germany	ready-to-use	HRP
AmpliStain™ anti-Rabbit 1-Step HRP (#AS-R1-HRP)	SDT GmbH, Baesweiler, Germany	ready-to-use	HRP
4′,6-diamidino-2-phenylindole (DAPI, #D9542-5MG)	Sigma, Hamburg, Germany	5 µg/mL	*w*/*o*
VECTASHIELD^®®^ Mounting Medium (#H-1000)	Vector Laboratories, Burlingame, CA, USA	ready-to-use	*w*/*o*
DAB Peroxidase Substrat Kit (#SK-4100)	Vector Laboratories, Burlingame, CA, USA	ready-to-use	DAB
Toluidine blue (Biovitrum, #07-002)	ErgoProduction LLC, Saint Petersburg, Russia	ready-to-use	*w*/*o*
Giemsa solution (Biovitrum, #21-023)	ErgoProduction LLC, Saint Petersburg, Russia	ready-to-use	*w*/*o*
Silver impregnation (Biovitrum, #21-026)	ErgoProduction LLC, Saint Petersburg, Russia	ready-to-use	*w*/*o*
Weigert for elastic fibers (Biovitrum, #21-030)	ErgoProduction LLC, Saint Petersburg, Russia	ready-to-use	*w*/*o*
Weiger—Van Gieson (Biovitrum, #21-020)	ErgoProduction LLC, Saint Petersburg, Russia	ready-to-use	*w*/*o*
Brilliant cresyl blue (Biovitrum, #20-041)	ErgoProduction LLC, Saint Petersburg, Russia	ready-to-use	*w*/*o*
Mayer’s hematoxylin (Biovitrum, #05-002)	ErgoProduction LLC, Saint Petersburg, Russia	ready-to-use	*w*/*o*

## Data Availability

All data and materials are available upon reasonable request to D.A. (email: atyakshin-da@rudn.ru) or O.M. (email: oleg.omedvedev@gmail.com).

## Supplementary Materials

The following supporting information can be downloaded at: https://www.mdpi.com/article/10.3390/ijms252011010/s1.
