# Supplementary material for: The Role of Mast Cells in the Remodeling Effects of Molecular Hydrogen on the Lung Local Tissue Microenvironment under Simulated Pulmonary Hypertension
Source: Int J Mol Sci. 2024 Oct 13;25(20):11010. doi: 10.3390/ijms252011010 (PMC11507233; doi:10.3390/ijms252011010)

**Supplementary S1.** Spatial phenotyping of tryptase in rat lung mast cells. Three-dimensional models of intracellular localization of tryptase. Technique: immunohistochemical tryptase staining. Nuclei are counterstained with DAPI. Control group. Secretory granules with different trypatase contents are located in separate loci of the mast cell. The peripheral localization of tryptase in secretory granules is clearly visible.

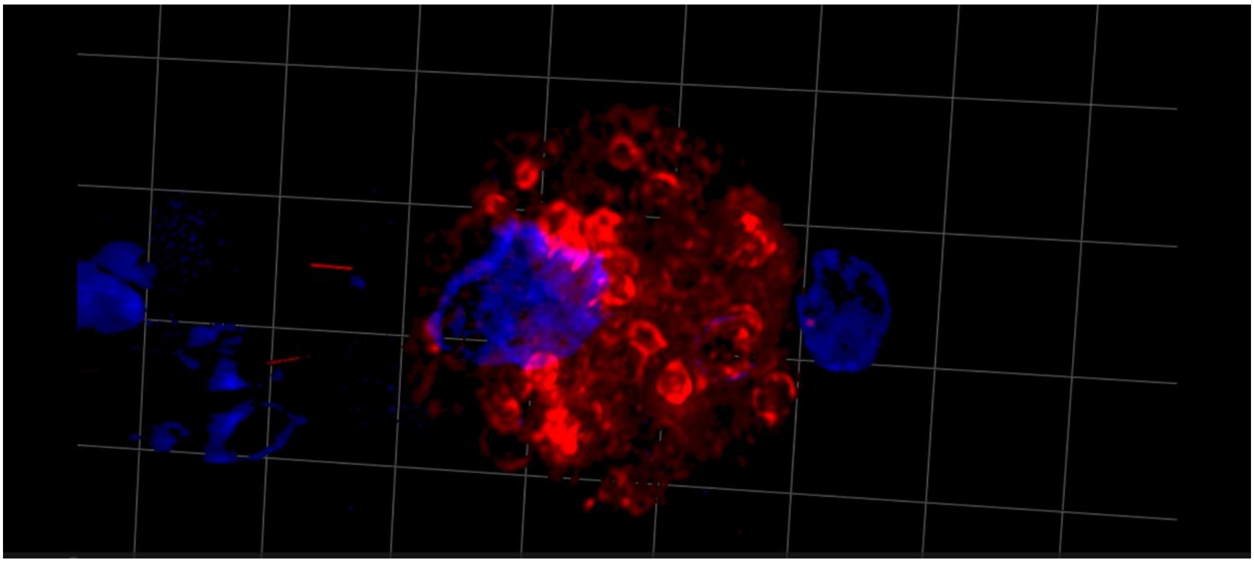

**Supplementary S2.** Spatial phenotyping of tryptase in rat lung mast cells. Three-dimensional models of intracellular localization of tryptase. Technique: immunohistochemical tryptase staining. Nuclei are counterstained with DAPI. Control group. Mast cells are located at a paracrine distance from each other.

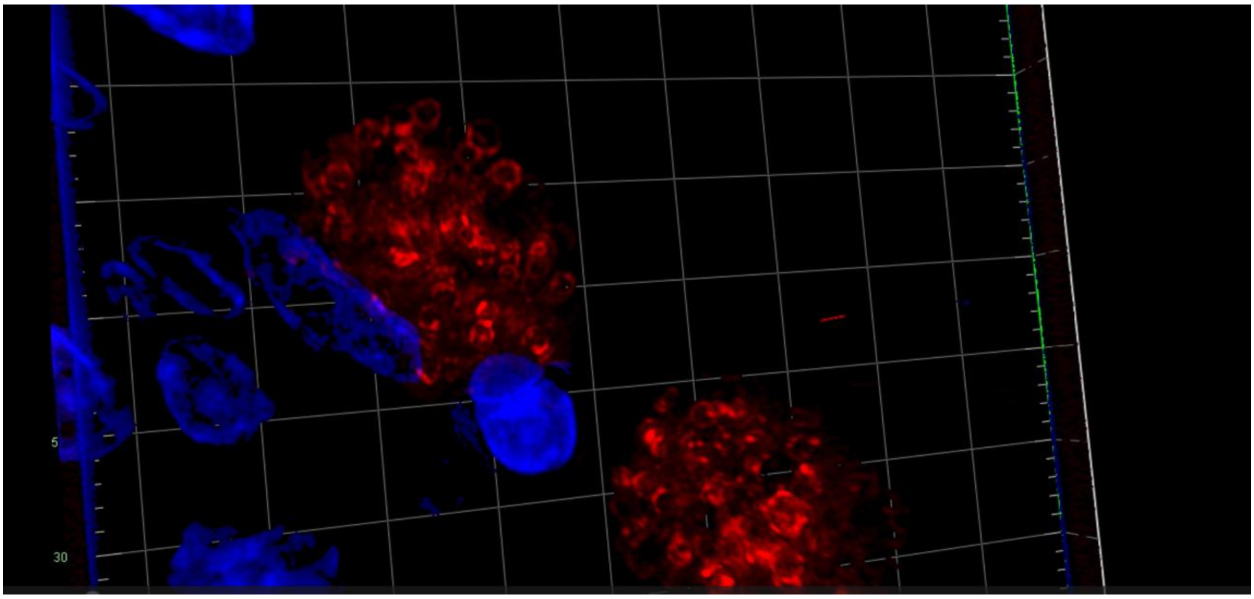

**Supplementary S3.** Spatial phenotyping of tryptase in rat lung mast cells. Three-dimensional models of intracellular localization of tryptase. Technique: immunohistochemical tryptase staining. Nuclei are counterstained with DAPI. MCT group. Interactions of mast cells with each other and other cells.

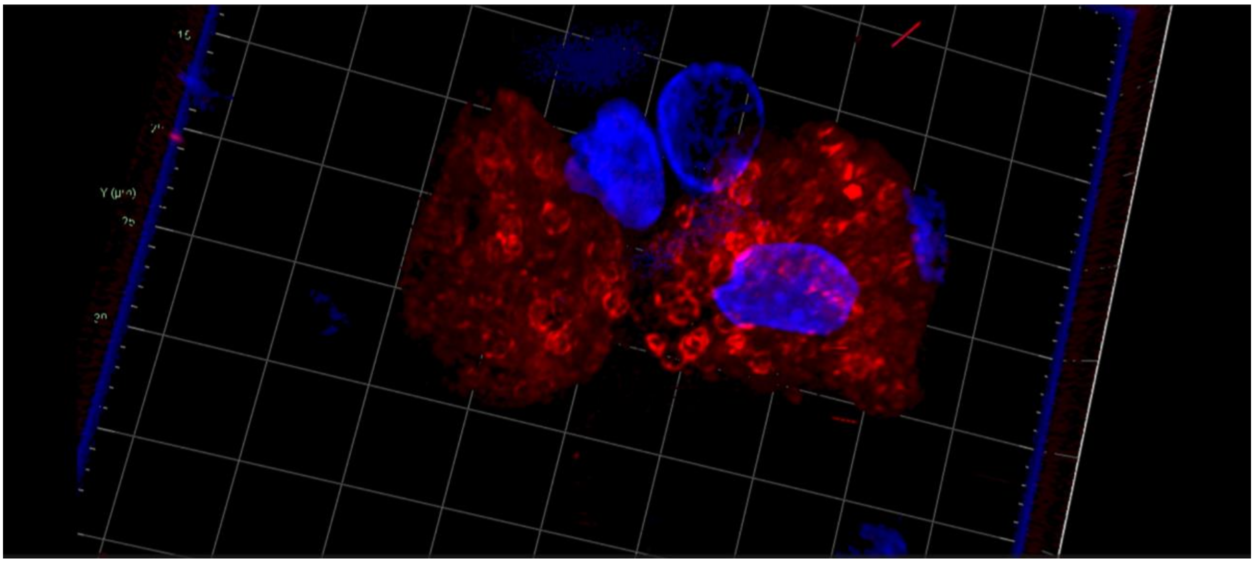

**Supplementary S4.** Spatial phenotyping of tryptase in rat lung mast cells. Three-dimensional models of intracellular localization of tryptase. Technique: immunohistochemical tryptase staining. Nuclei are counterstained with DAPI. Group of $H_2$ exposure. The cytoplasm is unevenly filled with tryptase-positive granules.

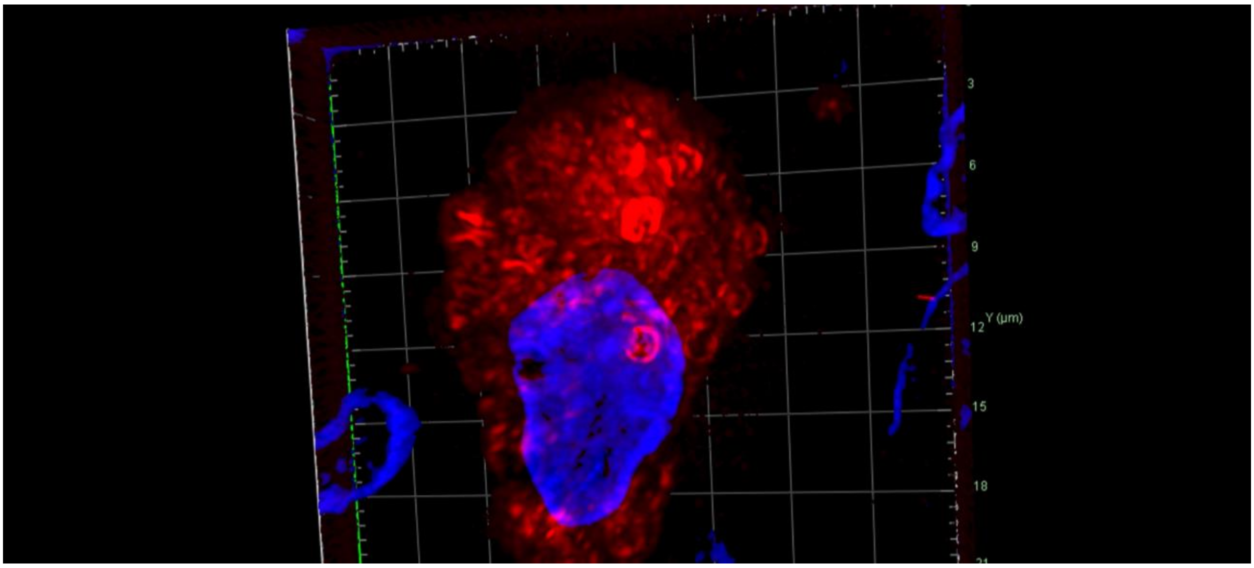

Supplement: Supplementary file 1 [file ijms-25-11010-s001.zip › ijms-3221879-supplementary.pdf]
